# Marine Oomycetes of the Genus *Halophytophthora* Harbor Viruses Related to Bunyaviruses

**DOI:** 10.3389/fmicb.2020.01467

**Published:** 2020-07-15

**Authors:** Leticia Botella, Josef Janoušek, Cristiana Maia, Marilia Horta Jung, Milica Raco, Thomas Jung

**Affiliations:** ^1^Phytophthora Research Centre, Department of Forest Protection and Wildlife Management, Faculty of Forestry and Wood Technology, Mendel University in Brno, Brno, Czechia; ^2^Biotechnological Centre, Faculty of Agriculture, University of South Bohemia, Ceske Budejovice, Czechia; ^3^Centre for Marine Sciences (CCMAR), University of Algarve, Faro, Portugal

**Keywords:** dsRNA, (-) ssRNA, RdRp, mycovirus, marine virology, estuaries

## Abstract

We investigated the incidence of RNA viruses in a collection of *Halophytophthora* spp. from estuarine ecosystems in southern Portugal. The first approach to detect the presence of viruses was based on the occurrence of dsRNA, typically considered as a viral molecule in plants and fungi. Two dsRNA-banding patterns (∼7 and 9 kb) were observed in seven of 73 *Halophytophthora* isolates tested (9.6%). Consequently, two dsRNA-hosting isolates were chosen to perform stranded RNA sequencing for *de novo* virus sequence assembly. A total of eight putative novel virus species with genomic affinities to members of the order *Bunyavirales* were detected and their full-length RdRp gene characterized by RACE. Based on the direct partial amplification of their RdRp gene by RT-PCR multiple viral infections occur in both isolates selected. Likewise, the screening of those viruses in the whole collection of *Halophytophthora* isolates showed that their occurrence is limited to one single *Halophytophthora* species. To our knowledge, this is the first report demonstrating the presence of negative (−) ssRNA viruses in marine oomycetes.

## Introduction

*Halophytophthora* species are fungal-like oomycetes with similar morphology and life cycles as members from their well-known plant pathogenic sister genus *Phytophthora* (Sullivan et al., 2018). As oomycetes they are distantly related to brown algae and belong to the Kingdom Stramenopila (Heterokonta). In pre-molecular times most marine oomycetes were assigned to the genus *Halophytophthora* but recent phylogenetic studies demonstrated that *Halophytophthora* is polyphyletic. Consequently, numerous *Halophytophthora* species were transferred to several new genera including *Calycofera*, *Phytopythium*, and *Salisapilia* (Marano et al., 2014; Bennett et al., 2017; Jung et al., 2017b; Bennett and Thines, 2019). Currently *Halophytophthora sensu stricto* contains nine described species. Most *Halophytophthora* species live in brackish and salt water habitats and have been traditionally described as saprophytes playing a key role as decomposers mainly in mangrove ecosystems (Newell and Fell, 1992, 1997). However, they may also be pathogenic. Several studies have already illustrated their pathogenicity on the marine eelgrass *Zostera marina* (Govers et al., 2016), which has a key ecological role along shores of North America and Eurasia (Man in ’t Veld et al., 2019). *Halophytophthora zostera* was shown to restrict the viability of *Z. marina* seeds and seedling development (Govers et al., 2016). Knowledge on *Halophytophthora* in temperate ecosystems has been scarce for a long time, hence, the conditions explaining why these decomposers may turn pathogenic are still unknown (Sullivan et al., 2018).

Virus diversity in marine ecosystems appears predominant (Suttle, 2005). With a total estimation of ∼10^30^ viruses, they have been implicated as the most abundant pathogens in the oceans (Suttle, 2005). Viruses infect all organisms from bacteria to whales but the majority infect bacteria (phages), which control microbial abundance and release dissolved organic matter, influencing global biogeochemical cycles (Wommack and Colwell, 2000; Weinbauer, 2004; Breitbart, 2012). Regarding lower eukaryotes, metagenomic and metatranscriptomic studies are rapidly expanding the knowledge about virus diversity and can detect the presence of a range of DNA or RNA genomes of various architectural type and size (Coy et al., 2018). Viruses with large double-stranded (ds) DNA genomes are typically found in phytoplankton (Weynberg et al., 2017). Single-stranded (ss) DNA viruses have been described in diatom algae (Nagasaki et al., 2005; Coy et al., 2018), corals (Vega Thurber et al., 2008) and stromatolites (Desnues et al., 2008). DsRNA viruses infect photosynthetic flagellates, heterotrophic protists (Desnues et al., 2008) and also fungi. Recently, dsRNA viruses hosted by marine fungi were isolated from the seagrass *Posidonia oceanica* (Nerva et al., 2015) and sea cucumber *Holothuria polii* (Nerva et al., 2019a), and identified a negative (−) ssRNA virus in *Penicillium roseopurpureum.*

The order *Bunyavirales* is comprised of viruses with segmented, linear (−) ssRNA genomes (Abudurexiti et al., 2019). The morphology of Bunyaviruses varies from symmetric icosahedral particles, similar to phleboviruses (Halldorsson et al., 2018), to spherical and pleomorphic particles, similar to orthobunyaviruses (Obijeski et al., 1976) and hantaviruses (Huiskonen et al., 2010). A model bunyavirus genome consists of three RNA segments, the smallest of which (S) may encode nucleocapside proteins (NP) and silencing suppressors (NS) while the medium sized (M) segment usually encodes glycoproteins (Gn and Gc) and movement proteins (Margaria et al., 2014). Finally, the largest segment (L) appears to possess a single open reading frame (ORF), which encodes the RNA dependent RNA polymerase (RdRp). According to the last update from the International Committee on Taxonomy of Viruses (ICTV), there are 12 bunyaviral families, four subfamilies, 46 genera and 287 species registered (Abudurexiti et al., 2019). However, due to more intensive samplings and, in particular, because of the use of *de novo* virus detection with metagenomics sequencing the diversity of bunyaviruses is constantly increasing (Li et al., 2015; Shi et al., 2016). Currently, bunyaviruses have been described in plants, invertebrate and vertebrate hosts and are transmitted by arthropod and mammalian vectors (Abudurexiti et al., 2019). Viruses with genomic similarities to bunyaviruses have also been described from diverse fungal hosts, including the phytopathogenic fungi *Botrytis cinerea* (Botrytis cinerea negative-stranded RNA virus 1, BcNSRV-1) (Donaire et al., 2016) and *Macrophomina phaseolina* (Macrophomina phaseolina negative-stranded RNA virus 1, MpNSRV1) (Marzano et al., 2016). Entoleuca phenui-like virus 1 (EnPLV1) has been identified from an avirulent isolate of *Entoleuca* sp. collected from avocado rhizosphere (Velasco et al., 2019). Coniothyrium diplodiella negative-stranded RNA virus 1 (CdNsRV1), Alternaria tenuissima negative-stranded RNA virus 2 (AtNsRV2) and Cladosporium cladosporioides negative-stranded RNA virus 2 (CcNsRV2), were isolated from the grapevine wood-inhabiting endophytes *Coniothyrium diplodiella*, *Alternaria tenuissima*, and *Cladosporium cladosporioides*, respectively (Nerva et al., 2019b). Likewise, the shiitake mushroom (*Lentinula edodes*) hosts Lentinula edodes negative-stranded virus 2 (LeNSRV2), which is related to phenuiviruses (Lin et al., 2019); a marine fungus, *P. roseopurpureum* hosts Penicillium roseopurpureum negative ssRNA virus 1 (PrNSRV1) (Nerva et al., 2019a) and the oomycete *Pythium polare*, Pythium polare bunya-like RNA virus 1 (PpBRV1) (Sasai et al., 2018). However, no effects of bunyavirus infection on growth or virulence of their fungal or oomycete hosts have been recorded yet.

Knowledge on viruses infecting oomycetes is relatively limited (Sutela et al., 2019). Several viruses have been described in downy mildews (biotrophic plant parasites) including unclassified (+) ssRNA viruses on *Sclerophthora macrospora* with similarities to a Noda-Tombus-like virus (Yokoi et al., 2003) and in *Plasmopara parasitica*, which causes hypovirulence on its host (Grasse et al., 2013; Grasse and Spring, 2017). The genus *Pythium* is comprised of water and soilborne oomycetes causing moderate to significant damages in plant roots (Sutela et al., 2019). Virus-like particles and/or dsRNA have been described in *Pythium irregular* (Gillings et al., 1993). Recently, an unclassified gammapartitivirus was reported in *Pythium nuun* (Shiba et al., 2018), a toti-like virus was also characterized from two strains of *Globisporangium splendens* (formerly *Pythium splendes*) and three virus-like sequences, Pythium polare RNA virus 1 (PpRV1), Pythium polare RNA virus 2 (PpRV2) and PpBRV1 were detected in *Pythium polare* infecting mosses in the Arctic (Sasai et al., 2018). In the genus *Phytophthora* the first virus to be reported in the USA (Hacker et al., 2005) was classified as an alphaendornavirus. It was found in an isolate of the undescribed *Phytophthora* taxon “douglasfir.” Later, similar virus strains were detected in *P. ramorum* isolates from several hosts in Europe (Kozlakidis et al., 2010) but no further investigations were performed. *Phytophthora infestans*, the causal agent of potato late blight, also harbors four RNA viruses. Phytophthora infestans RNA virus 1 and 2, (PiRV-1 and PiRV-2, respectively) apparently represent novel virus families (Cai et al., 2009, 2019), Phytophthora infestans RNA virus 3 (PiRV-3) is clustered with the newly proposed family “*Fusagraviridae*” (Cai et al., 2013) and Phytophthora infestans RNA virus 4 (PiRV-4) is an unclassified member of *Narnaviridae* (Cai et al., 2012). Recently, PiRV2, known to be 100% transmittable through asexual spores, was shown to stimulate sporangia production and enhance the virulence of *P. infestans* (Cai et al., 2019). Phytophthora cactorum RNA virus 1 (PcRV1), a toti-like virus, has been newly described in an isolate of *Phytophthora cactorum* collected from a trunk lesion on silver birch in Denmark (Poimala and Vainio, 2020).

Because the genus *Halophytophthora* represents a unique group of marine microorganisms with uncertain roles as phytopathogens on coastal and estuarine grasses, we wished to expand current research to better understand their behavior by investigating their potential virome. More precisely, our study had two goals: (i) to confirm the presence of viruses in these marine oomycetes by combining traditional and state-of-art technologies, and (ii) to assess their abundance and genetic variability in a collection of *Halophytophthora* isolates from Portugal.

## Materials and Methods

### Oomycete Isolates

All *Halophytophthora* isolates studied were collected from seven localities in southern Portugal (Table 1) using an *in situ* baiting technique (Jung et al., 2017a). At each site, 15–20 non-wounded young leaves of three tree species, *Ceratonia siliqua*, *Citrus sinensis* and *Quercus suber*, were placed as baits in a 25 × 30 cm raft, prepared using fly mesh and styrofoam, and the raft put to float in the tidal zone. The rafts were collected after 3 days. Baiting leaves were washed in distilled water and blotted dry on filter paper. Five to ten pieces (approximately 2 × 2 mm) were cut from the margins of each watersoaked or necrotic lesion of each leaf, blotted on filter paper and plated onto selective PARPNH agar (V8-juice agar (V8A) amended with 10 μg/ml pimaricin, 200 μg/ml ampicillin, 10 μg/ml rifampicin, 25 μg/ml pentachloronitrobenzene (PCNB), 50 μg/ml nystatin and 50 μg/ml hymexazol). The *Halophytophthora* isolates are part of the collection of the Phytophthora Research Centre (Mendel University), located in Brno, Czech Republic. The Supplementary Table S1 provides detailed information and species identification of all *Halophytophthora* isolates, which is currently under investigation. For the different purposes of the present study all isolates were grown for 7–21 days in darkness on V8A media, covered with cellophane (EJA08-100; Gel Company, Inc., CA, United States).

**TABLE 1:** Relation of isolates per sampling locality and virus detection.

**Locality, municipality**	**N^&^**	**dsRNA screening^$^**	**dsRNA patterns**	**RT-PCR screening^+^**	Virus found by RT-PCR*
Ribeira de Odelouca, Silves	14	14	∼9 kb	14	None
Rio Séqua, Tavira	9	9	None	9	None
Parque Natural da Ria Formosa, Santa Luzia, Tavira	24	18	∼7 and 9 kb	21	HRV1-8
Parque Natural da Ria Formosa, Quelfes, Olhão	6	5	∼9 kb	6	None
Ria de Alvor, Alvor, Portimão	8	6	None	8	None
Parque Natural da Ria Formosa, Almancil, Loulé	9	8	∼9 kb	8	None
Sapal de Castro Marim/Rio Guadiana, Castro Marim	32	13	None	29	None

**^&^**Total number of isolates screened for viruses by both dsRNA isolation and partial amplification of their RdRp by RT-PCR using specific primers for each of them; **^$^**number of isolates used in the dsRNA screening; **^+^**number of isolates screened by RT-PCR; *viruses detected by RT-PCR. See Supplementary Table S1 for the complete information of the isolates, DsRNA and RT-PCR screening.

### DsRNA Isolation

**FIGURE 1:**
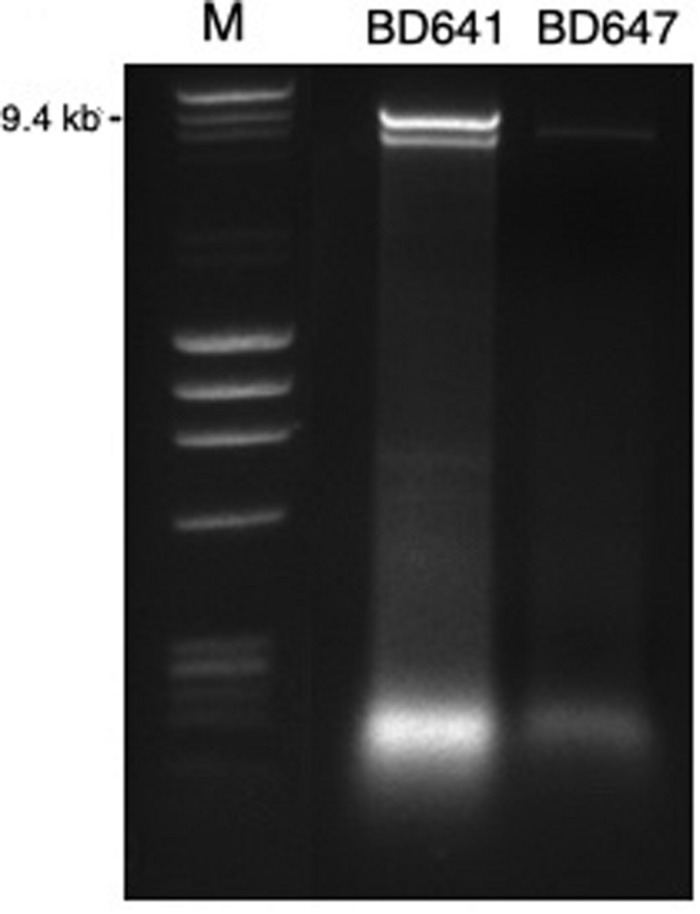
DsRNA profiles found in *Halophytophthora* isolates BD641 (1) and BD647 (2). DsRNA bands were visualized by gel electrophoresis (120 V; 90 min). Analyzed fragments were separated on 1.5% agarose gel prepared with a TBE buffer (106177; Merck KGaA, Germany) and stained by Ethidium Bromide (E1510, Sigma-Aldrich, Germany). Ready-to-use DNA size and mass standard (F-303SD, Thermo Scientific).

A total of 73 isolates from Portugal were screened for dsRNA (Figure 1), which was purified using a modified version of the protocol of Morris and Dodds (1979). Approximately 2 g of fresh mycelium were transferred to a 50 ml Falcon tube and disrupted by vortexing for 3 min with two stainless steel balls (diameter 10 mm). The rest of the protocol was performed as described by Tuomivirta et al. (2002).

### Stranded RNA Sequencing of Samples BD641 and BD647

Total RNA was purified from approximately 50 mg of fresh mycelium using SPLIT RNA Extraction Kit (Lexogen, Austria) and treated with DNase I (ThermoFisher Scientific). RNA quantity and quality were checked using respectively a Qubit^®^ 2.0 Fluorometer (Invitrogen) and Tape Station 4200 (Agilent) resulting in a RNA integrity number (RIN) of 10.

Approximately 1 μg of total RNA eluted in RNase-free water was sent to Fasteris SA (Plan-les-Ouates, Switzerland) for RNA library construction and deep sequencing. The library preparation was performed using the Illumina TruSeq^®^ stranded RNA Sample Preparation Kit (Illumina, San Diego, CA, United States). The library was sequenced in pair-end (2x 75 nt) runs on an Illumina NextSeq 500 machine. Prior to the library preparation, Ribo-Zero rRNA Removal Kit (Human/Mouse/Rat) was used successfully. An “in-lane” PhiX control spike was included in each lane of the flow-cell. 93.94% of the reads had a quality value (Q30) ≥ 30, i.e., less than 1 error in 1000 bases. The raw data was deposited in the Sequence Read Archive (SRA) with the BioProject ID PRJNA619952 and BioSample ID SAMN14856044.

### Bioinformatics Analysis

Adapter trimming was not needed as the adapter sequence was only present in less than 1% of the reads. All reads were compared to the genome sequence of *Phytophthora cinnamomi*^1^ with BWA 0.7.5a^2^. Unmapped reads were selected from the mapping files and saved in a fastq format. BAM post-processing was performed with toolbox for manipulation of SAM/BAM files V. 1.1^3^ and BEDTOOLS V. 2.21.0^4^. *De novo* assembly was performed with VELVET V1.2.10^5^. As the repeat resolution module of VELVET assumes linearity and uniform coverage distribution, it produces fragmented transcriptome assemblies. To consider the unequal expression levels and alternative splicing breakpoints, the preliminary assemblies produced by VELVET were inputted to OASES 0.2.08^6^, which exploits read sequence and pairing information (if available) to produce transcript isoforms. When possible, OASES also detects and reports standard alternative splicing events. The first 1M paired-reads of each library were mapped on each OASES assembly. The alignment was done using the mapping software BWA 0.7.5a and SAMTOOLS 1.1^7^. Reads mapping to several positions on the reference sequence with the same mapping quality were attributed at random to one of the positions with a mapping quality of 0. When an input read had N’s in their nucleotide sequence, BWA replaced the Ns with a random nucleotide. *De novo* assembly and validation mapping were done using BWA 0.7.5a. The result of the mapping of the reads on the reference sequences is summarized in Supplementary Table S2. The assemblies with a hash of 51 gave the highest representability. The numbers of reads aligning and coverage depth for the final viral sequence were calculated using Geneious Prime^®^ 2020.0.4.

The sequence alignment and blast output post-processing with BLAST ncbi-blast-2.2.26^7^. The contig files were aligned to the NCBI database using a local installation of the BLAST software. The results from the BLASTn, BLASTX and the BLAST search on the viral reference dataset (RefSeq) were compared. The output file was then parsed using in-house (Fasteris) scripts.

### Rapid Amplification of cDNA Ends (RACE) and Full-Length Viral Sequences

In order to determine the 5′- and 3′- terminal sequences and lengths of the putative viral genomes the SMARTer^®^ RACE 5′/3′ KIT (Takara Bio USA, Inc.) was used with total RNA extracted as described above using SPLIT RNA Extraction Kit (Lexogen, Austria). The 3′ Poly(A) Tailing of RNA was performed at 37°C for 20 min using yeast Poly(A) Polymerase (MCLAB, San Francisco, CA). Thereafter, approximately 1 μg of total RNA was used for RACE First-strand cDNA synthesis of both 5′- and 3′- termini was performed using specific primers designed for the eight putative viruses in a 5′- and 3′ – orientation (Supplementary Table S3) as described by the manufacturer. Then, 5′-RACE and 3′-RACE PCR amplification was performed to generate the corresponding cDNA fragments using SeqAmp DNA Polymerase (Takara Bio USA, Inc.) as described by the manufacturer. Amplicons were extracted and purified from the gel using NucleoSpin^®^ Gel and PCR Clean-Up Kit (Macherey-Nagel GmbH & Co. KG). All amplicons were cloned using In-Fusion HD Cloning Kit and Stellar Competent Cells (Takara Bio USA, Inc.). Recombinant plasmids were extracted with Thermo Scientific GeneJET Plasmid Miniprep Kit and sequenced by GATC Biotech, Germany. Nested PCR was often necessary in order to precisely amplify the virus 3′ termini. Here, the screening forward primers (Supplementary Table S3) in combination with the Universal Primer mix (UPM) from the SMARTer^®^ RACE 5′/3′ KIT (Takara Bio USA, Inc.) were used to generate amplicons to act as template for nested PCR. The nested PCR RACE primers and the Short Universal primer provided by the kit were used. To obtain the 3′ end terminal sequence of virus 8 a different primer was designed.

### Screening for Virus Incidence by RT-PCR Amplification

Mycelium from individual isolates was collected and homogenized using a bead tube holder devide (740469; Macherey-Nagel; Germany). RNA was isolated and purified using the Monarch Total RNA Miniprep Kit (T2010S; New England Biolabs, MA, United States). Efficient RNA extraction was achieved using the manufacturer’s recommended protocol for tough-to-lyse samples. Contaminating host DNA was removed from the extracts using a combination of gDNA removal columns and DNase I treatment. RNA was eluted in 30 μl volumes and stored at −80°C.

cDNA was synthesized using the ProtoScript II First Strand cDNA Synthesis Kit (E6560; New England Biolabs, MA, United States). Here, oligo d(T)23VN was incubated with the RNA at 65°C for 5 min. Next, random primer mix was added together with ProtoScript II Enzyme and Reaction Mix, followed by incubation at 25°C for 5 min, 42°C for 60 min and denatured at 80°C for 5 min.

Virus incidence in the *Halophytophthora* ssp. isolates was tested using PCR amplification with virus-specific primers (Supplementary Table S4). All primers were used to amplify a fragment of the RdRp gene of each virus and were designed by Primer 3 2.3.7 under Geneious Prime^®^ 2020.0.4. Primer sequences, amplicon sizes and annealing temperatures are shown in Supplementary Table S4. PCR amplification was performed with 12.5 μl OneTaq Quick-Load 2X Master Mix with Standard Buffer (M0486; New England Biolabs, MA, United States), 0.5 μl of each 10 mM primer, 3 μl of cDNA in a total volume of 25 μl. Cycling conditions were used according to manufacturer’s recommendations and the annealing temperature of each primer set was calculated using the on-line tool^8^ (v1.9.9 May 30).

Amplicons were visualized and separated by electrophoresis (300 V; 10 min) through 1.5% agarose gels in TBE buffer (106177; Merck KGaA, Germany) and with DNA Stain G (39803; SERVA; Germany). Amplicons of the expected lengths were purified and sequenced in both directions by GATC BioTech (Eurofins; Konstanz, Germany) with the primers used in the initial PCR amplification. All the amplicon sequences were deposited in the GenBank under the accession number MT277331-349. As an internal control for successful PCR amplification from viral RNA templates routine amplification of an actin housekeeping gene (Weiland and Sundsbak, 2000), was performed simultaneously with primers MIDFWACT and MIDREVACT (Supplementary Table S4) in all experiments under identical conditions (Supplementary Figure S1).

### Genetic Variability and Phylogenetic Analysis

Pairwise identities of the nucleotide and amino acid sequences (Supplementary Tables S5, S6) were obtained after aligning the eight viral nucleotide and amino acid sequences by MUSCLE (Edgar, 2004) and calculated using Geneious Prime^®^ 2020.0.4. DnaSP v5 (Rozas et al., 2017) was used to estimate genetic diversity parameters for strains of the different *Halophytophthora* viruses.

**FIGURE 2:**
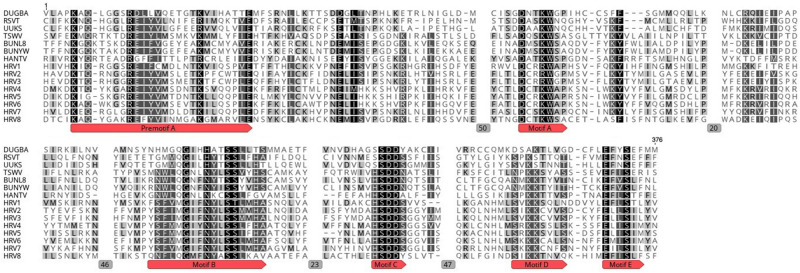
Amino acid alignment showing conserved motifs A to E and premotif A within the RdRp of HRV1-8 and selected bunyaviruses. DUGBA, Dugbe virus (accession number Q66431.1); RSVT, Rice stripe virus (Q85431.1); UUKS, Uukuniemi virus S23 (P33453.1); TSWV, Tomato spotted wilt virus (P28976.1); BUNYW, Bunyawera virus (P20470); BUNL8, La Crosse virus L78 (Q8JPR2.1); HANTV, Hantaan virus 76-118 (P23456.3). Gray boxes with numbers represent the number of positions deleted in the MUSCLE alignment.

In order to search for conserved domains within the putative viral proteins the NCBI CD-search tool was used^9^ (Lu et al., 2020). Viral protein sequences were aligned by MUSCLE (Edgar, 2004) using Geneious Prime^®^ 2020.0.4 (Figure 2).

**FIGURE 3:**
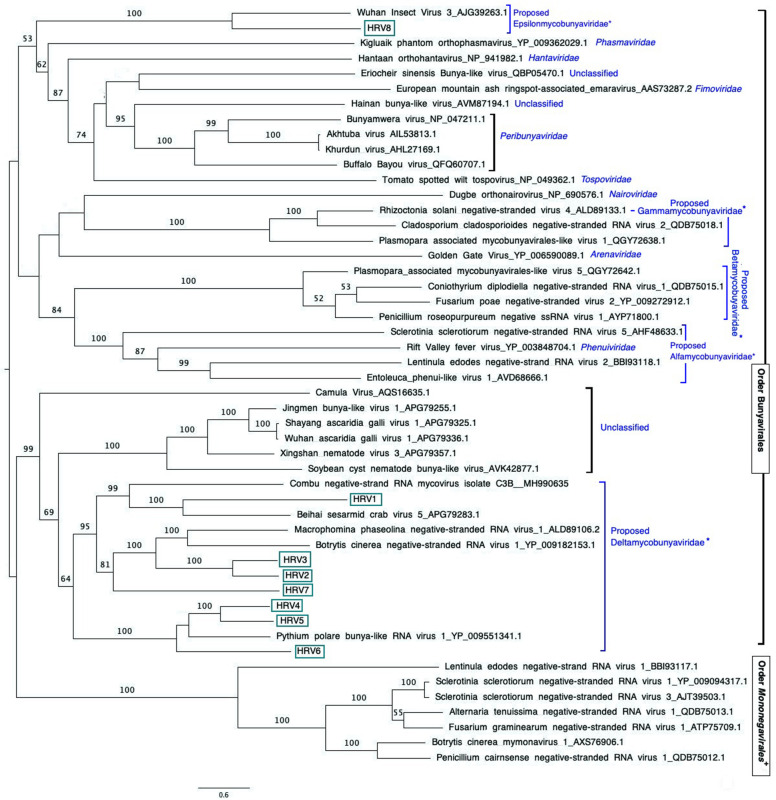
Maximum likelihood tree (RAxML) depicting the phylogenetic relationship of the predicted RdRp of *Halophytophthora* viruses with other complete RdRp belonging to related (–) ssRNA viruses from the orders *Bunyavirales* and *Mononegavirales*. Nodes are labeled with bootstrap support values. Branch lengths are scaled to the expected underlying number of amino acid substitutions per site. Nodes are labeled only with bootstrap percentages ≥ 50%. Tree is rooted in the midpoint. Halophytophthora RNA viruses 1-8 (HRV1-8) are represented by their abbreviation names. Family classification and the corresponding pBLAST accession numbers are shown next to the virus names. *Family names proposed by Nerva et al. (2019b) and family Epsilonmycobunyaviridae, proposed in this study. **^+^**These members of the Order *Mononegavirales* are classified within the family *Mymonaviridae.*

A maximum likelihood phylogenetic tree was constructed using a rapid bootstrapping algorithm (Stamatakis et al., 2008) in RAxML-HPC v.8 on XSEDE conducted in CIPRES Science Gateway (Miller et al., 2010) (Figure 3). Tree search was enabled under the GAMMA model to avoid thorough optimization of the best scoring ML tree at the end of the run. The Jones–Taylor–Thornton (JTT) model was chosen as the substitution model for proteins. Bootstrapping was configurated with the recommended parameters provided by CIPRES Science Gateway. The resulting data were visualized using the software FIGTREE software version 1.4.4^10^.

## Results

### Identification of the Mycoviruses Infecting Selected *Halophytophthora* Isolates

A total of 7 out of 73 *Halophytophthora* isolates (9.6 %) from three different Portuguese localities were found to contain one or two dsRNA segments as illustrated by gel electrophoresis (Table 1 and Supplementary Table S1). Those isolates which contained dsRNA apparently belong to three different previously undescribed *Halophytophthora* species (Supplementary Table S1). Isolates BD093 and BD094 (*Halophytophthora* sp. 01) from Ribeira de Odelouca (Silves) contained a ∼9 kb-dsRNA segment. Isolates BD641, BD647 and BD654 (*H*. sp. 04) from Parque Natural da Ria Formosa (Tavira) contained two different banding patterns with ∼7 and/or ∼9 kb dsRNA bands (Figure 1). BD665 (*H*. sp. 03) from Parque Natural da Ria Formosa in Olhão and BD685 from Parque Natural da Ria Formosa in Almancil, Loulé, both contained a ∼7 kb segment. Two of these positive isolates, BD641 and BD647, were chosen for stranded RNA sequencing.

One sequencing library was prepared from rRNA-depleted total RNA and generated 3.6 × 10^11^ 75-nucleotide (nt) paired end (PE) sequence reads (Supplementary Table S2). When they were assembled against the genome of *P. cinamomi* as host reference sequence and 1.15 × 10^8^ PEs mapped. The unmapped 1.12 × 10^8^ PE reads were selected for the further investigation. The final contig file had 35,621 contigs (including 548 undetermined bases) with an average length of 894 nt and a maximum contig length of 21,602 nt. Assembly of the sequence reads from the final contig file revealed eight potential virus sequences as evidenced by significant E-values and identity percentage of their predicted amino acid sequences (Table 2). The contigs representing the eight potential viruses had different read numbers and coverage (Table 3), HRV8 had the deepest coverage and HRV2 the least. The eight sequences identified were similar to members of the order *Bunyavirales* hosted by fungal pathogens, including *Botrytis cinerea* (Botrytis cinerea negative-stranded RNA virus 1, BcNSRV-1, YP_009182153), *Macrophomina phaseolina* (Macrophomina phaseolina negative-stranded RNA virus 1, MpNSRV1, ALD89106.2), oomycetes, such as *Pythium polare* (Pythium polare bunya-like RNA virus 1, PpRV1, YP_09551341.1) and arthropod-like insects including *Asellus* sp. (Wuhan insect virus 3, AJG39263.1) and crabs (Beihai sesarmid crab virus 5, APG79283.1) (Table 2). According to the convention of the International Committee on Taxonomy of Viruses (ICTV) the eight putative viruses were designated as Halophytophthora RNA virus (HRV) 1–8 (Table 2).

**TABLE 2:** Identification of *Halophytophthora* viruses’ most similar RdRp sequences in the GenBank based on BLASTX search.

**Virus name**	**Acronym**	**GenBank accession numbers**	**Most similar virus in GenBank**	***E* value**	**Query cover (%)**	**Identity (%)**
Halophytophthora RNA virus 1	HRV1	MT277350	Beihai sesarmid crab virus 5	0.0	72	32.73
Halophytophthora RNA virus 2	HRV2	MT277351	Botrytis cinerea negative-stranded RNA virus 1	0.0	52	32.40
Halophytophthora RNA virus 3	HRV3	MT277352	Botrytis cinerea negative-stranded RNA virus 1	0.0	54	32.13
Halophytophthora RNA virus 4	HRV4	MT277353	Pythium polare bunya-like RNA virus 1	0.0	82	38.35
Halophytophthora RNA virus 5	HRV5	MT277354	Pythium polare bunya-like RNA virus 1	0.0	84	37.84
Halophytophthora RNA virus 6	HRV6	MT277355	Pythium polare bunya-like RNA virus 1	0.0	86	38.30
Halophytophthora RNA virus 7	HRV7	MT277356	Macrophomina phaseolina negative-stranded RNA virus 1	7e^–126^	68	28.76
Halophytophthora RNA virus 8	HRV8	MT277357	Wuhan Insect virus 3	0.0	60	58.07

**TABLE 3:** Parameters of the genome organization of *Halophytophthora* viruses and their initial contigs.

	**Initial contig length (nt)**	**Genome size (nt)**	**Largest ORF (nt)**	**Largest ORF (aa)**	**Function**	**Read Number**	**Coverage**
Halophytophthora RNA virus 1	9,341	9,340	8,184	2,728	RdRp	630,934	5,066
Halophytophthora RNA virus 2	9,134	9,152	8,361	2,787	RdRp	15,298	126
Halophytophthora RNA virus 3	9,113	9,184	8,985	2,995	RdRp	716,385	5,850
Halophytophthora RNA virus 4	7,794	7,822	7,716	2,572	RdRp	341,627	3,275
Halophytophthora RNA virus 5	7,103	7,735	6,681	2,227	RdRp	376,150	3,647
Halophytophthora RNA virus 6	6,326	7,742	5,796	1,932	RdRp	843,893	8,175
Halophytophthora RNA virus 7	5,644	6,789	6,705	2,235	RdRp	264,248	2,919
Halophytophthora RNA virus 8*	3,299	7,874	7,788	2,596	RdRp	720,641	6,864

*The length of the 3′ end remains unsure.

### Virus Genome Organization

Based on our analyses, only ORFs encoding an RdRp gene were found.

**FIGURE 4:**
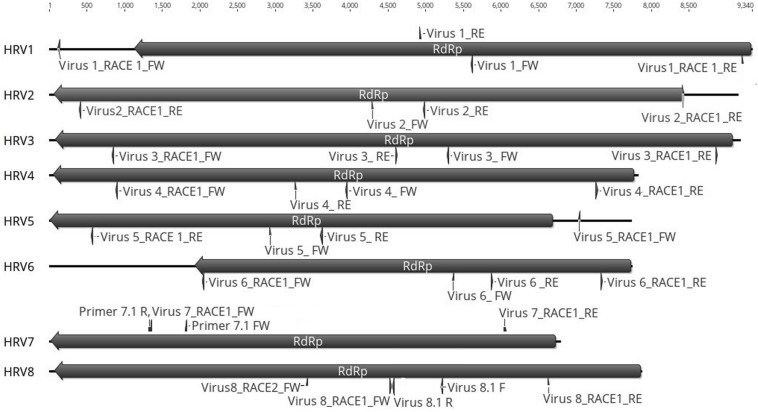
Representation of the RdRp organization of the eight viruses found in *Halophytopthora* spp. Boxes represent the ORFs detected and they are shown in the direction of the original contig, from which primers for RT-PCR screening and RACE were designed. Primer names are indicated next to their position in the nucleotide sequence, primers were designed and named based on the orientation of the original stranded contig, which in the case of HRV3 and 5 it was genomic and in the case of HRV1, 2, 4, 6, 7, and 8, it was antigenomic. Numbers represent the genome length.

Based on the sequence of the original contigs oligonucleotide primers were designed to amplify and confirm the terminal RdRp sequences of the eight viruses (Supplementary Table S4) some of which, including HRV 1, 2, 3, 4, and 5, were complete or nearly complete. The remainder of the viral terminal sequences of HRV6, 7, and 8 were completed by RACE. The eight virus genomes ranged in size from 7.8 up to 9.3 kb (Table 3) including a single large ORF encoding an RdRp flanked by 5′ and 3′-untranslated regions (UTRs) (Figure 4). The longest genome corresponds to HRV1 (9,340 bp) and the shortest to HRV6 (6,816 bp) (Figure 3 and Table 3).

Based on the amino acid (aa) sequence analysis of the eight viral ORFs all of them contained conserved regions belonging to pfam04196, Bunyavirus RdRp, which is the solitary member of the superfamily cl20265. Taken in turn, HRV1 has a conserved region ranging from aa 1454–1759 (expect value 1.01 e^– 11^); HRV2 from aa 1036–1394 (*E*-value: 9.37 e^–13^); HRV3 from aa 1239–1588 (*E*-value: 5.11 e^–10^); HRV4 from aa 735–1208 (*E*-value: 1.51 e^–07^); HRV5 from aa 716–1301 (*E*-value: 1.53 e^–07^); HRV6 from aa 1132- 1421 (*E*-value: 4.89 e^–06^); HRV7 from aa 1204-1771 (*E*-value: 6.24e^–10^); HRV8 from aa 894 to 1515 (*E*-value: 1.27 e^–30^) and from aa 615 to 806 (*E*-value: 7.55 e^–06^). The alignment of the RdRps of HRV1-8 with other viral sequences indicated the presence of the typical conserved motifs in RdRps of bunyaviruses (Kormelink et al., 2011) with certain variability (Figure 2): motif A (DxxxWx), where HRV1-3 had an arginine (R) instead of lysine (K) before the tryptophan (W), motif B (XGxxNxxSS), motif C (SDD), motif D (KK) and motif E (ExxSx). Premotif A with the three basic residues inside (K, R and R/K) and, downstream, the glutamic acid (E), were also identified. In addition, the conserved aa triplet TPD, typical of bunyaviruses (Muller et al., 1994), was identified in HRV1-7 (but not in HRV8) in positions 160, 107, 321, 83, 87, 282, and 160, respectively. The doublet RY was strictly conserved in HRV1, 4-8 in positions 894, 754, 757, 970, 1212 and 894, respectively, and partly conserved in HRV2 and 3 (positions 205 and 1019, respectively), which had R (arginine) and N (asparagine) instead of Y (tyrosine).

### Phylogenetic Relationships Between the *Halophytophthora* Viruses and Other (−) ssRNA Viruses

An examination of the phylogenetic relationships between the *Halophytophthora* viruses and other (−) ssRNA viruses retrieved from the GenBank shows that they cluster with unclassified viruses with genomic affinities of the order *Bunyavirales* (Figure 3). HRV1 to 7 are very closely related to one another and form a cluster with MpNSRV1, BcNSRV-1 and PpRV1. This cluster includes other bunyaviruses described in invertebrate species representing different metazoan phyla including sesarmid crabs (Beihai sesarmid crab virus 5) and nematodes (Soybean cyst nematode bunya-like virus 1, SCN-BLV1). HRV8 differs from HRV1-7 and is grouped in a different cluster with a virus apparently hosted by *Aselus* sp. insects (Wuhan insect virus 3), which are phylogenetically closer to reported members of bunyavirus families including *Tospoviridae*, *Fimoviridae*, *Peribunyaviridae*, *Hantaviridae*, and *Phenuiviridae*. All HRV1-8 appeared distanced from members of the family *Mymonaviridae* in the order *Mononegavirales*.

### HRV Occurrence in the *Halophytophthora* Isolate Collection

A total of four out of 95 *Halophytophthora* isolates (4.2%) from one sampling locality (Parque Natural da Ria Formosa, Santa Luzia, Tavira) resulted to host one or more of the eight *Halophytophthora* viruses described in this study. The most abundant was HRV6, which was present in 4 isolates (BD641, BD647, BD650, and BD654); HRV1 and HRV8 in 3 isolates (BD641, BD647, and BD654). HRV7 in isolates BD641 and BD647; HRV3 in isolates BD641 and BD654; HRV5 is in isolates BD647 and BD654, and HRV2 and HRV4 seem to be only present in isolate BD647.

While isolates BD093, BD094, BD665, and BD685 contained dsRNA elements (results not resulted) they were unrelated to the bunyaviral dsRNAs described as determined by RT-PCR (Supplementary Table S1). In some cases, e.g., HRV6 in BD650, dsRNA elements were not visualized by gel staining but RT-PCR confirmed the presence of a virus (Supplementary Table S1). Isolates BD641 and BD647 contained two and one dsRNA elements, respectively (Table 1 and Figure 1), which have not been correlated with HRV1-8 by RT-PCR using dsRNA as a template.

### Genetic Diversity of HRV

At the nucleotide level (Supplementary Table S5), the highest pairwise identity between viruses was HRV2 and 3 (57.05%) followed by HRV2 and 3 (39.00%). Conversely, the lowest pairwise identity was between HRV1 and 7 (23.43%). At the amino acid (aa) level (Supplementary Table S6), HRV2 and 3 also have the highest pairwise identity (47.48%), followed by HRV5 and 4 (39.91%). And, HRV7 and 8, the lowest (9.00%).

The genetic variability of the partially sequenced RdRp genes of the eight *Halophytophthora* virus strains was also assessed for those viruses that were hosted in more than one isolate (Table 4). All of the isolates appear to have very high haplotype diversity with HRV5 possessing the highest nucleotide diversity (0.12) and segregating sites (80). Conversely, HRV7 seems to be the least genetically diverse virus with a low nucleotide diversity (0.05) and the lowest number of segregating sites (34).

**TABLE 4:** Genetic parameters of the partial RdRp nucleotide sequences of HRV strains.

**Species**	***N***	**Sites**	**Net sites**	**S**	**h**	**Hd**	π	**AvNumDif**
HRV1	3	708	704	51	3	1.00	0.05	34
HRV2	1	–	–	–	–	–	–	–
HRV3	2	701	700	52	2	1.00	0.07	52
HRV4	1	–	–	–	–	–	–	–
HRV5	2	655	654	80	2	1.00	0.12	80
HRV6	4	523	523	50	4	1.00	0.05	28
HRV7	3	473	473	34	3	1.00	0.05	23
HRV8	3	659	652	65	3	1.00	0.07	44

*(–) not assessed; (N) Number of isolates; (Sites) length of the sequence, (Net sites) length of the sequence analyzed; (S) number of segregating sites; (h) number of haplotypes; (π), nucleotide diversity estimated by the average number of differences per site between two sequences; (AvNumDif) average number of differences.*

## Discussion

Traditional dsRNA extraction procedures were used to identify the potential presence of viruses in a collection of *Halophytophthora* spp. isolates from estuarine ecosystems in southern Portugal. Subsequently, stranded RNA sequencing followed by *de novo* contig assembling and comparative BLAST searches were used to identify eight putative novel viral sequences in two dsRNA-hosting isolates (Figure 1) belonging to the same *Halophytophthora* species (*H.* sp. 04, Supplementary Table S1). Primer-specific RT-PCR and 5′ and 3′ RACE were performed to confirm the presence and complete the sequences of each virus. All eight viral sequences contained elements of RdRp genes and bunyavirus motifs (Figure 2). Their eight virus genomes ranged in size from 6.8 to 9.3 kb (Table 3). BLAST analysis revealed that the eight viruses were significantly similar in sequence (*ca*. 30% identity; Table 2) to a number of unclassified bunya-like viruses isolated from different fungi, oomycetes, insects and crabs (Table 2). HRV8 differs from HRV1-7 and appears in a phylogenetic outgroup and is 58.07% similar to Wuhan insect virus 3 which is apparently hosted by *Asellus* sp. in China.

The overall pairwise nucleotide and aa identity of the eight viruses was 30.7 and 16.6%, respectively. HRV2 and 3 appear to one another apparently share identities of 57.05 and 47.80% at the nucleotide and aa levels, respectively. The remainder of the pairwise sequence comparisons (PASC) between the HRV isolates reveal lower identities of 40 and 20% at the nucleotide and aa levels (Supplementary Tables S5, S6). Analysis of the RdRp of HRV1-8 illustrate that they differ > 10% between one another and with their most similar matches in the GenBank. Since the primary classification criteria for genus and species used currently are based on pairwise sequence comparisons (PASC) and phylogenetic analyses, the viruses discovered in this investigation may constitute eight novel virus species, designated as Halophytophthora RNA Virus 1-8 (HRV1-8).

Bunyaviruses are enveloped viruses with a genome consisting of three ssRNA segments (called L, M, and S), the S RNA encodes the nucleocapsid protein, the M glycoproteins and the L segments encode the RNA polymerase. Each genome segment is coated by the viral nucleoproteins (NPs) and the polymerase (L protein) to form a functional ribonucleoprotein (RNP) complex, which is necessary for the RNA replication and gene transcription (reviewed in Ferron et al., 2017). However, in our study we have only discovered the L segment. Our result does not categorically rule out the existence of M and S segments but their copy number might be very low compared to the polymerase fragment. The NGS performed in this investigation may not have analyzed sufficient reads to identify smaller HRV genomic components but this is unlikely because it has been established that 100 M reads is sufficient to identify all RNAs of interest and the rRNA depletion worked successfully. A quality check for the presence of rRNAs was performed by mapping the reads on rRNA animal and human databases and <5% of rRNA reads were detected. Similar to HRV1-8 a number of other bunya-like mycoviruses, including BcNSRV-1 (Donaire et al., 2016), MpNSRV1 (Marzano et al., 2016), and PpRV1 (Sasai et al., 2018) apparently only possess an RdRp. However, it is more plausible that the genome description of these viruses (including HRV1-8) is incomplete. The putative NP and other non-structural (Ns) associated proteins are likely not conserved enough to be detected by homology, in contrast to what has been observed in other viruses including PrNSRV1 (Nerva et al., 2019a) and LeNSRV2 (Lin et al., 2019).

The phylogenetic tree shown in Figure 2, which includes HRV1-8 and 42 (−) ssRNA viruses, illustrates that HRV1-7 cluster with several unclassified viruses found in a variety of fungal, oomycete, nematode, crab and insect hosts, and a virus detected by NGS approach from the stool of a rhesus monkey (*Macaca mulatta*) (Zhao et al., 2017). More specifically, HRV4-6 are significantly similar in sequence to PpRV1 from the Arctic and Antarctic moss pathogen *Pythium polare* (Sasai et al., 2018). HRV2, 3, and 7 are closer to BcNSRV-1 from the air-borne fungal pathogen *Botrytis cinerea* (Donaire et al., 2016) and MpNSRV1 from the soil-borne pathogen *Macrophomina phaseolina* (Marzano et al., 2016), and HRV1 is grouped with Combu negative-strand RNA mycovirus isolate C3B from the soil fungus *Mucor irregularis* in Belém, a port municipality in the Brasilian Amazon (accession number MH990635, unpublished), and Beihai sesarmid crab virus 5 detected by NGS from a sesarmid crab mix from Beihai, China. Sesarmid crabs have an important ecological role in mangrove ecosystems because they consume large amounts of leaf litter (Lee, 1998). HRV8 has some sequence similarity with classified bunyaviruses and, particularly, with Wuhan insect virus 3, hosted by an individual of *Asellus* sp. This genus of isopod crustaceans is known to feed primarily on decaying vegetation, microscopic algae and small invertebrates (reviewed in O’Callaghan et al., 2019). Since *Halophytophthora* spp. are the first colonizers of fallen mangrove leaves (Newell and Fell, 1992; Man in ’t Veld et al., 2019) and estuarine grasses due to their ability to produce large amounts of chemotactic zoospores (reviewed in Man in ’t Veld et al., 2019) they likely share their habitat with insects, nematodes and crustaceans, where they undoubtedly interact. As an example, the class *Oomycota* includes marine holocarpic pathogens of nematodes, algae, crustaceans and molluscs (Thines and Choi, 2016), thus some of the viruses discovered by NGS of invertebrates may have originated from their gut mycoflora and/or parasites. Even though arthropods are thought to be the ancestral hosts of bunyaviruses (Marklewitz et al., 2015) if viruses with similar genomes continue to be discovered in other invertebrates, protists and fungi (Shi et al., 2018) this theory may have to be redefined.

The phylogenetic tree shown in Figure 2 has similarities with previous phylogenetic studies that group novel bunya-like mycoviruses separated from most of the currently classified bunyavirus families (Donaire et al., 2016; Marzano et al., 2016; Sasai et al., 2018; Nerva et al., 2019b). And, it also supports the proposal of different mycobunyaviral families (Nerva et al., 2019b): family Deltamycobunyaviridae with MpNSRV1, BcNSRV1 also includes PpRV1 and HRV1-7; two other families (proposed alfamycobunyaviridae and betamycobunyaviridae) including myco-phlebo-like viruses (Lin et al., 2019; Velasco et al., 2019; Chiapello et al., 2020) and the family Gammamycobunyaviridae with CcNSRV2, RsNSV4 and Plasmopara associated mycobunyavirales-like RNA Virus 2 (Nerva et al., 2019b; Chiapello et al., 2020). In addition, we propose a fifth family (Epsilonmycobunyaviridae) for HRV8 and Wuhan insect virus 3. As indicated previously (Nerva et al., 2019b), this classification is not formal as it has not been accepted by ICTV but it sheds light on the current knowledge of novel bunya-like mycoviruses.

The genetic variability of the partial RdRp sequences of HRV1-8 showed that they have a relatively low nucleotide but high haplotype diversity since all the strains were different (Table 4). In this regard, HRV5 possessed the highest nucleotide variability (0.12). It is well established that bunyavirus replication is error-prone and results in genome modification. Such modifications can accumulate over the time either due either to random genetic drift or as genetic adaptations of the virus to a new environment and/or a new host (Schneider and Roossinck, 2001; Li and Roossinck, 2004; Barr and Fearns, 2010). Novel viral genotypes can be generated through mutation, recombination and reassortment. Viral reassortment seems to be a powerful mechanism underlying the evolution of the *Bunyavirales* order (Coupeau et al., 2019). Briese et al. (2013) pointed out that most bunyaviruses described so far are actually reassortants of existing or extinguished viruses. This process leads to the generation of progeny viruses with novel genomic organizations as a result of gene shuffling between coinfecting closely related bunyaviruses (Coupeau et al., 2019). The same might exist for HRV1-8, which are only hosted by four *Halophytophthora* isolates, BD641, BD647, BD650 and BD654. These isolates belong to the same species (*H.* sp. 04) and were sampled from the same site, Parque Natural da Ria Formosa, Santa Luzia, Tavira, Portugal. As this yet undescribed *Halophytophthora* species has a functional homothallic sexual system it is feasible that the viruses were transferred between isolates during the mating process or by simple contact between vegetative hyphae, resulting in the coinfection of their viruses. Interestingly, HRV1-8 only occur in *H.* sp. 04 despite the presence of at least seven *Halophytophthora* species at the Santa Luzia site. The viruses were not found in isolates of *H.* sp. 04 at the Ria de Alvor, Alvor, Portimão site, suggesting that HRV1-8 might only be transmitted intraspecifically between co-occurring *Halophytophthora* isolates. Little is known about virus transmission in oomycetes. The only studies concerning virus transmission in oomycetes were performed in *P. infestans*, where several viruses are stably maintained (Cai et al., 2019). For instance, individual zoospores show 100% inheritance of PiRV-3 (Cai et al., 2019). In addition, PiRV-2 is readily horizontally transmitted by hyphal anastomosis, and vertically transmitted by asexual reproduction through sporangia (Cai et al., 2019). However, attempts to transfer PiRV-2 into apparently vegetative incompatible *P. infestans* isolates failed. Although several studies have demonstrated that interspecies transmission does occur naturally (Van Diepeningen et al., 1998; Melzer et al., 2002; Vainio et al., 2011), mycovirus transmission typically occurs between strains of the same fungal species. Viruses spread readily through fungal hyphal networks crossing pores contained in compartmenting septa. However, as potential detrimental cytoplasmic elements, their transmission between strains may be restrained by a genetic self/non-self recognition system termed vegetative incompatibility (*vic*), mating type incompatibility or intersterility (Leslie and Zeller, 1996). Oomycetes lack septa and in some cases, as in the genus *Phytophthora*, they are prompted to interspecific hybridizations which play a major role in speciation and species radiations in diverse natural ecosystems (Schardl and Craven, 2003; Jung et al., 2017a). However, viruses might also be recognized as invasive compounds of the cytoplasmatic entities limiting their transfer to another species. While *vic* and mating systems serve as antiviral defense mechanisms at the population level, RNA silencing or RNA interference (RNAi) provides a fungal antiviral defense response at the cellular level (Nuss, 2011). Antiviral RNA silencing has been demonstrated in different types of fungi including the chestnut pathogen *Cryphonectria parasitica* (Segers et al., 2007) or the arbuscular mycorrhizal fungus *Gigaspora margarita* (Silvestri et al., 2020) but also in oomycetes (Fahlgren et al., 2013). Since the RNAi machinery targets possible detrimental non-self-nucleic acids, virus-infected host organisms are normally enriched with viral small interfering (si) RNA. For instance, in an analysis of the virus-derived small RNAs following high-throughput sequencing of the *Halophytophthora* isolate BD647 small RNA reads were mapped to HRV6 (unpublished data).

## Conclusion

A combination of traditional and new technologies has been used to identify and sequence eight bunya-like mycoviruses that coinfect isolates belonging to the same species of *Halophytophthora* from southern Portuguese estuaries. However, any relationships between the different viruses and any effects of the viruses on the phenotype, virulence and host range of their oomycete host remain unknown.

## Data Availability Statement

The datasets presented in this study can be found in online repositories. The names of the repository/repositories and accession number(s) can be found at: https://www.ncbi.nlm.nih.gov/bioproject/PRJNA619952.

## Author Contributions

LB and TJ: original idea. CM, TJ, and MJ: sampling and phylogenetic characterization of *Halophytophthora* isolates. LB, JJ, and MR: methodology. LB: formal analysis, writing—original draft preparation. LB, TJ, and JJ: writing—review and editing.

## Conflict of Interest

The authors declare that the research was conducted in the absence of any commercial or financial relationships that could be construed as a potential conflict of interest.

## Footnotes

1 ftp://ftp.ncbi.nlm.nih.gov/genomes/all/GCA/001/314/365/GCA_001314365.1_MP94-48v2/GCA_001314365.1_MP94-48v2_genomic.fna.gz

2 http://bio-bwa.sourceforge.net

3 http://www.htslib.org/

4 http://code.google.com/p/bedtools/

5 https://www.ebi.ac.uk/~zerbino/velvet/

6 https://www.ebi.ac.uk/~zerbino/oases/

7 http://www.htslib.org/doc/samtools-11.html

8 http://tmcalculator.neb.com/

9 https://www.ncbi.nlm.nih.gov/Structure/cdd/wrpsb.cgi

10 http://tree.bio.ed.ac.uk/software/figtree/

11 http://www.ncbi.nlm.nih.gov/BLAST

## Supplementary Material

TABLE S1 Complete list of isolates used in the present study, indicating species identification, location, dsRNA pattern and results of the RT-PCR screening title.

TABLE S2 Mapping results for *P. cinnamomi* as reference sequence.

TABLE S3 Gene-Specific Primers (GSPs) used in the SMARTer RACE reactions.

TABLE S4 Specific primers used for the partial amplification of the viral RdRp in order to screen the presence of each virus in all the isolates of the collection.

TABLE S5 Percentages of pairwise identities between the eight viral full-length nucleotide genome sequences.
