# Supplementary material for: Marine Oomycetes of the Genus Halophytophthora Harbor Viruses Related to Bunyaviruses
Source: Front Microbiol. 2020 Jul 15;11:1467. doi: 10.3389/fmicb.2020.01467 (PMC7375090; doi:10.3389/fmicb.2020.01467)
Supplement: TABLE S1 — Complete list of isolates used in the present study, indicating species identification, location, dsRNA pattern and results of the RT-PCR screening title. [file Table_1.docx]

**Table S1.** Complete list of *Halophytophthora* isolates used in the present study, indicating species identification, sampling location, dsRNA pattern and results of the RT-PCR screening.

|  | **Species** | **Sampling locality** | **DsRNA** | **Virus 1** | **Virus 2** | **Virus 3** | **Virus 4** | **Virus 5** | **Virus 6** | **Virus 7** | **Virus 8** | **Actine** |
| --- | --- | --- | --- | --- | --- | --- | --- | --- | --- | --- | --- | --- |
| **BD084** | *Halophytophthora avicenniae* | Ribeira de Odelouca, Silves | 0 | 0 | 0 | 0 | 0 | 0 | 0 | 0 | 0 | Y |
| **BD085** | *H. avicenniae* | Ribeira de Odelouca, Silves | 0 | 0 | 0 | 0 | 0 | 0 | 0 | 0 | 0 | Y |
| **BD086** | *H. avicenniae* | Ribeira de Odelouca, Silves | 0 | 0 | 0 | 0 | 0 | 0 | 0 | 0 | 0 | Y |
| **BD087** | *H. avicenniae* | Ribeira de Odelouca, Silves | 0 | 0 | 0 | 0 | 0 | 0 | 0 | 0 | 0 | Y |
| **BD088** | *H. avicenniae* | Ribeira de Odelouca, Silves | 0 | 0 | 0 | 0 | 0 | 0 | 0 | 0 | 0 | Y |
| **BD089** | *H. avicenniae* | Ribeira de Odelouca, Silves | 0 | 0 | 0 | 0 | 0 | 0 | 0 | 0 | 0 | Y |
| **BD090** | *H.* sp. 01 | Ribeira de Odelouca, Silves | 0 | 0 | 0 | 0 | 0 | 0 | 0 | 0 | 0 | Y |
| **BD091** | *H.* sp. 01 | Ribeira de Odelouca, Silves | 0 | 0 | 0 | 0 | 0 | 0 | 0 | 0 | 0 | Y |
| **BD092** | *H.* sp. 01 | Ribeira de Odelouca, Silves | 0 | 0 | 0 | 0 | 0 | 0 | 0 | 0 | 0 | Y |
| **BD093** | *H.* sp. 01 | Ribeira de Odelouca, Silves | 9 kb | 0 | 0 | 0 | 0 | 0 | 0 | 0 | 0 | Y |
| **BD094** | *H.* sp. 01 | Ribeira de Odelouca, Silves | 9 kb | 0 | 0 | 0 | 0 | 0 | 0 | 0 | 0 | Y |
| **BD095** | *H. avicenniae* | Ribeira de Odelouca, Silves | 0 | 0 | 0 | 0 | 0 | 0 | 0 | 0 | 0 | Y |
| **BD875** | *H.* sp. 01 | Ribeira de Odelouca, Silves | 0 | 0 | 0 | 0 | 0 | 0 | 0 | 0 | 0 | Y |
| **BD876** | *H.* sp. 01 | Ribeira de Odelouca, Silves | 0 | 0 | 0 | 0 | 0 | 0 | 0 | 0 | 0 | Y |
| **BD628** | *H.* sp. 02 | Rio Séqua, Tavira | 0 | 0 | 0 | 0 | 0 | 0 | 0 | 0 | 0 | Y |
| **BD630** | *H.* sp. 01 | Rio Séqua, Tavira | 0 | 0 | 0 | 0 | 0 | 0 | 0 | 0 | 0 | Y |
| **BD631** | *H.* sp. 01 | Rio Séqua, Tavira | 0 | 0 | 0 | 0 | 0 | 0 | 0 | 0 | 0 | Y |
| **BD632** | *H.* sp. 02 | Rio Séqua, Tavira | 0 | 0 | 0 | 0 | 0 | 0 | 0 | 0 | 0 | Y |
| **BD633** | *H. avicenniae* | Rio Séqua, Tavira | 0 | 0 | 0 | 0 | 0 | 0 | 0 | 0 | 0 | Y |
| **BD635** | *H. avicenniae* | Rio Séqua, Tavira | 0 | 0 | 0 | 0 | 0 | 0 | 0 | 0 | 0 | Y |
| **BD636** | *H. avicenniae* | Rio Séqua, Tavira | 0 | 0 | 0 | 0 | 0 | 0 | 0 | 0 | 0 | Y |
| **BD637** | *H.* sp. 01 | Rio Séqua, Tavira | 0 | 0 | 0 | 0 | 0 | 0 | 0 | 0 | 0 | Y |
| **BD638** | *H.* sp. 02 | Rio Séqua, Tavira | 0 | 0 | 0 | 0 | 0 | 0 | 0 | 0 | 0 | Y |
| **BD639** | *H.* sp. 06 | Parque Natural da Ria Formosa, Santa Luzia, Tavira | 0 | 0 | 0 | 0 | 0 | 0 | 0 | 0 | 0 | Y |
| **BD640** | *H.* sp. 05 | Parque Natural da Ria Formosa, Santa Luzia, Tavira | 0 | 0 | 0 | 0 | 0 | 0 | 0 | 0 | 0 | Y |
| **BD641** | *H.* sp. 04 | Parque Natural da Ria Formosa, Santa Luzia, Tavira | 7-9 Kb | 1 | 1 | 1 | 1 | 0 | 1 | 1 | 1 | Y |
| **BD642** | *H.* sp. 06 | Parque Natural da Ria Formosa, Santa Luzia, Tavira | 0 | 0 | 0 | 0 | 0 | 0 | 0 | 0 | 0 | Y |
| **BD643** | *H.* sp. 06 | Parque Natural da Ria Formosa, Santa Luzia, Tavira | 0 | 0 | 0 | 0 | 0 | 0 | 0 | 0 | 0 | Y |
| **BD644** | *H.* sp. 07 | Parque Natural da Ria Formosa, Santa Luzia, Tavira | n.a. | 0 | 0 | 0 | 0 | 0 | 0 | 0 | 0 | Y |
| **BD645** | *H.* sp. 06 | Parque Natural da Ria Formosa, Santa Luzia, Tavira | n.a. | 0 | 0 | 0 | 0 | 0 | 0 | 0 | 0 | Y |
| **BD646** | *H.* sp. 08 | Parque Natural da Ria Formosa, Santa Luzia, Tavira | 0 | 0 | 0 | 0 | 0 | 0 | 0 | 0 | 0 | Y |
| **BD647** | *H.* sp. 04 | Parque Natural da Ria Formosa, Santa Luzia, Tavira | 7 Kb | 1 | 0 | 0 | 1 | 1 | 1 | 1 | 1 | Y |
| **BD649** | *H.* sp. 06 | Parque Natural da Ria Formosa, Santa Luzia, Tavira | 0 | 0 | 0 | 0 | 0 | 0 | 0 | 0 | 0 | Y |
| **BD650** | *H.* sp. 04 | Parque Natural da Ria Formosa, Santa Luzia, Tavira | 0 | 0 | 0 | 0 | 0 | 0 | 1 | 0 | 0 | Y |
| **BD651** | *H.* sp. 01 | Parque Natural da Ria Formosa, Santa Luzia, Tavira | 0 | 0 | 0 | 0 | 0 | 0 | 0 | 0 | 0 | Y |
| **BD652** | *H.* sp. 01b | Parque Natural da Ria Formosa, Santa Luzia, Tavira | 0 | 0 | 0 | 0 | 0 | 0 | 0 | 0 | 0 | Y |
| **BD653** | *H.* sp. 01b | Parque Natural da Ria Formosa, Santa Luzia, Tavira | 0 | 0 | 0 | 0 | 0 | 0 | 0 | 0 | 0 | Y |
| **BD654** | *H.* sp. 04 | Parque Natural da Ria Formosa, Santa Luzia, Tavira | 9 kb | 1 | 0 | 1 | 0 | 1 | 1 | 0 | 1 | Y |
| **BD655** | *H.* sp. 04 | Parque Natural da Ria Formosa, Santa Luzia, Tavira | 0 | 0 | 0 | 0 | 0 | 0 | 0 | 0 | 0 | Y |
| **BD656** | *H.* sp. 05 | Parque Natural da Ria Formosa, Santa Luzia, Tavira | n.a. | 0 | 0 | 0 | 0 | 0 | 0 | 0 | 0 | Y |
| **BD881** | *H.* sp. 06 | Parque Natural da Ria Formosa, Santa Luzia, Tavira | 0 | 0 | 0 | 0 | 0 | 0 | 0 |  | 0 | Y |
| **BD882** | *H.* sp. 06 | Parque Natural da Ria Formosa, Santa Luzia, Tavira | 0 | n.a. | n.a. | n.a. | n.a. | n.a. | n.a. | n.a. | n.a. | Y |
| **BD885** | *H.* sp. 08 | Parque Natural da Ria Formosa, Santa Luzia, Tavira | 0 | n.a. | n.a. | n.a. | n.a. | n.a. | n.a. | n.a. | n.a. | Y |
| **BD888** | *H.* sp. 07 | Parque Natural da Ria Formosa, Santa Luzia, Tavira | 0 | n.a. | n.a. | n.a. | n.a. | n.a. | n.a. | n.a. | n.a. | Y |
| **BD889** | *H.* sp. 07 | Parque Natural da Ria Formosa, Santa Luzia, Tavira | n.a. | 0 | 0 | 0 | 0 | 0 | 0 | 0 | 0 | Y |
| **BD890** | *H.* sp. 07 | Parque Natural da Ria Formosa, Santa Luzia, Tavira | n.a. | 0 | 0 | 0 | 0 | 0 | 0 | 0 | 0 | Y |
| **BD891** | *H.* sp. 07 | Parque Natural da Ria Formosa, Santa Luzia, Tavira | n.a. | 0 | 0 | 0 | 0 | 0 | 0 | 0 | 0 | Y |
| **BD657** | *H.* sp. 03 | Parque Natural da Ria Formosa, Quelfes, Olhão | 0 | 0 | 0 | 0 | 0 | 0 | 0 | 0 | 0 | Y |
| **BD658** | *H.* sp. 07 | Parque Natural da Ria Formosa, Quelfes, Olhão | 0 | 0 | 0 | 0 | 0 | 0 | 0 | 0 | 0 | Y |
| **BD659** | *H.* sp. 06 | Parque Natural da Ria Formosa, Quelfes, Olhão | 0 | 0 | 0 | 0 | 0 | 0 | 0 | 0 | 0 | Y |
| **BD660** | *H.* sp. 07 | Parque Natural da Ria Formosa, Quelfes, Olhão | n.a. | 0 | 0 | 0 | 0 | 0 | 0 | 0 | 0 | Y |
| **BD664** | *H.* sp. 06 | Parque Natural da Ria Formosa, Quelfes, Olhão | 0 | 0 | 0 | 0 | 0 | 0 | 0 | 0 | 0 | Y |
| **BD665** | *H.* sp. 03 | Parque Natural da Ria Formosa, Quelfes, Olhão | 9 kb | 0 | 0 | 0 | 0 | 0 | 0 | 0 | 0 | Y |
| **BD668** | *H.* sp. 01 | Ria de Alvor, Alvor, Portimão | 0 | 0 | 0 | 0 | 0 | 0 | 0 | 0 | 0 | Y |
| **BD669** | *H.* sp. 01 | Ria de Alvor, Alvor, Portimão | n.a. | 0 | 0 | 0 | 0 | 0 | 0 | 0 | 0 | Y |
| **BD670** | *H. avicenniae* | Ria de Alvor, Alvor, Portimão | 0 | 0 | 0 | 0 | 0 | 0 | 0 | 0 | 0 | Y |
| **BD671** | *H. avicenniae* | Ria de Alvor, Alvor, Portimão | 0 | 0 | 0 | 0 | 0 | 0 | 0 | 0 | 0 | Y |
| **BD673** | *H.* sp. 01 | Ria de Alvor, Alvor, Portimão | 0 | 0 | 0 | 0 | 0 | 0 | 0 | 0 | 0 | Y |
| **BD674** | *H.* sp. 01 | Ria de Alvor, Alvor, Portimão | 0 | 0 | 0 | 0 | 0 | 0 | 0 | 0 | 0 | Y |
| **BD675** | *H.* sp. 04 | Ria de Alvor, Alvor, Portimão | 0 | 0 | 0 | 0 | 0 | 0 | 0 | 0 | 0 | Y |
| **BD676** | *H.* sp. 04 | Ria de Alvor, Alvor, Portimão | n.a. | 0 | 0 | 0 | 0 | 0 | 0 | 0 | 0 | Y |
| **BD679** | *H.* sp. 02 | Parque Natural da Ria Formosa, Almancil, Loulé | 0 | 0 | 0 | 0 | 0 | 0 | 0 | 0 | 0 | Y |
| **BD680** | *H.* sp. 03 | Parque Natural da Ria Formosa, Almancil, Loulé | 0 | n.a. | n.a. | n.a. | n.a. | n.a. | n.a. | n.a. | n.a. | n.a. |
| **BD681** | *H.* sp. 02 | Parque Natural da Ria Formosa, Almancil, Loulé | n.a. | 0 | 0 | 0 | 0 | 0 | 0 | 0 | 0 | N |
| **BD682** | *H. avicenniae* | Parque Natural da Ria Formosa, Almancil, Loulé | 0 | 0 | 0 | 0 | 0 | 0 | 0 | 0 | 0 | Y |
| **BD683** | *H.* sp. 01 | Parque Natural da Ria Formosa, Almancil, Loulé | 0 | 0 | 0 | 0 | 0 | 0 | 0 | 0 | 0 | Y |
| **BD684** | *H.* sp. 01 | Parque Natural da Ria Formosa, Almancil, Loulé | 0 | 0 | 0 | 0 | 0 | 0 | 0 | 0 | 0 | Y |
| **BD685** | *H.* sp. 01 | Parque Natural da Ria Formosa, Almancil, Loulé | 7 kb | 0 | 0 | 0 | 0 | 0 | 0 | 0 | 0 | Y |
| **BD686** | *H.* sp. 02 | Parque Natural da Ria Formosa, Almancil, Loulé | 0 | 0 | 0 | 0 | 0 | 0 | 0 | 0 | 0 | Y |
| **BD687** | *H. avicenniae* | Parque Natural da Ria Formosa, Almancil, Loulé | 0 | 0 | 0 | 0 | 0 | 0 | 0 | 0 | 0 | Y |
| **BD689** | *H. avicenniae* | Sapal de Castro Marim / Rio Guadiana, Castro Marim | 0 | 0 | 0 | 0 | 0 | 0 | 0 | 0 | 0 | Y |
| **BD690** | *H. avicenniae* | Sapal de Castro Marim / Rio Guadiana, Castro Marim | 0 | 0 | 0 | 0 | 0 | 0 | 0 | 0 | 0 | Y |
| **BD692** | *H.* sp. 01 | Sapal de Castro Marim / Rio Guadiana, Castro Marim | 0 | 0 | 0 | 0 | 0 | 0 | 0 | 0 | 0 | Y |
| **BD693** | *H.* sp. 01 | Sapal de Castro Marim / Rio Guadiana, Castro Marim | 0 | 0 | 0 | 0 | 0 | 0 | 0 | 0 | 0 | Y |
| **BD695** | *H.* sp. 09 | Sapal de Castro Marim / Rio Guadiana, Castro Marim | 0 | n.a. | n.a. | n.a. | n.a. | n.a. | n.a. | n.a. | n.a. | n.a. |
| **BD696** | *H.* sp. 07 | Sapal de Castro Marim / Rio Guadiana, Castro Marim | 0 | 0 | 0 | 0 | 0 | 0 | 0 | 0 | 0 | Y |
| **BD697** | *H.* sp. 01 | Sapal de Castro Marim / Rio Guadiana, Castro Marim | 0 | 0 | 0 | 0 | 0 | 0 | 0 | 0 | 0 | Y |
| **BD698** | *H. avicenniae* | Sapal de Castro Marim / Rio Guadiana, Castro Marim | n.a. | 0 | 0 | 0 | 0 | 0 | 0 | 0 | 0 | Y |
| **BD841** | *H.* sp. 01 | Sapal de Castro Marim / Rio Guadiana, Castro Marim | 0 | 0 | 0 | 0 | 0 | 0 | 0 | 0 | 0 | Y |
| **BD842** | *H. avicenniae* | Sapal de Castro Marim / Rio Guadiana, Castro Marim | 0 | 0 | 0 | 0 | 0 | 0 | 0 | 0 | 0 | Y |
| **BD843** | *H. avicenniae* | Sapal de Castro Marim / Rio Guadiana, Castro Marim | 0 | 0 | 0 | 0 | 0 | 0 | 0 | 0 | 0 | Y |
| **BD844** | *H. avicenniae* | Sapal de Castro Marim / Rio Guadiana, Castro Marim | n.a. | 0 | 0 | 0 | 0 | 0 | 0 | 0 | 0 | Y |
| **BD845** | *H. avicenniae* | Sapal de Castro Marim / Rio Guadiana, Castro Marim | 0 | n.a. | n.a. | n.a. | n.a. | n.a. | n.a. | n.a. | n.a. | n.a. |
| **BD898** | *H. avicenniae* | Sapal de Castro Marim / Rio Guadiana, Castro Marim | n.a. | 0 | 0 | 0 | 0 | 0 | 0 | 0 | 0 | Y |
| **BD899** | *H.* sp. 01 | Sapal de Castro Marim / Rio Guadiana, Castro Marim | n.a. | 0 | 0 | 0 | 0 | 0 | 0 | 0 | 0 | Y |
| **BD900** | *H.* sp. 01 | Sapal de Castro Marim / Rio Guadiana, Castro Marim | n.a. | 0 | 0 | 0 | 0 | 0 | 0 | 0 | 0 | Y |
| **BD901** | *H.* sp. 01 | Sapal de Castro Marim / Rio Guadiana, Castro Marim | n.a. | 0 | 0 | 0 | 0 | 0 | 0 | 0 | 0 | Y |
| **BD902** | *H.* sp. 01 | Sapal de Castro Marim / Rio Guadiana, Castro Marim | n.a. | 0 | 0 | 0 | 0 | 0 | 0 | 0 | 0 | Y |
| **BD903** | *H.* sp. 01 | Sapal de Castro Marim / Rio Guadiana, Castro Marim | n.a. | 0 | 0 | 0 | 0 | 0 | 0 | 0 | 0 | Y |
| **BD904** | *H.* sp. 01 | Sapal de Castro Marim / Rio Guadiana, Castro Marim | n.a. | 0 | 0 | 0 | 0 | 0 | 0 | 0 | 0 | Y |
| **BD907** | *H. avicenniae* | Sapal de Castro Marim / Rio Guadiana, Castro Marim | n.a. | 0 | 0 | 0 | 0 | 0 | 0 | 0 | 0 | Y |
| **BD908** | *H.* sp. 07 | Sapal de Castro Marim / Rio Guadiana, Castro Marim | n.a. | 0 | 0 | 0 | 0 | 0 | 0 | 0 | 0 | Y |
| **BD909** | *H. avicenniae* | Sapal de Castro Marim / Rio Guadiana, Castro Marim | n.a. | 0 | 0 | 0 | 0 | 0 | 0 | 0 | 0 | Y |
| **BD914** | *H.* sp. 01 | Sapal de Castro Marim / Rio Guadiana, Castro Marim | 0 | 0 | 0 | 0 | 0 | 0 | 0 | 0 | 0 | Y |
| **BD915** | *H.* sp. 01 | Sapal de Castro Marim / Rio Guadiana, Castro Marim | 0 | n.a. | n.a. | n.a. | n.a. | n.a. | n.a. | n.a. | n.a. | n.a. |
| **BD916** | *H.* sp. 01 | Sapal de Castro Marim / Rio Guadiana, Castro Marim | n.a. | 0 | 0 | 0 | 0 | 0 | 0 | 0 | 0 | Y |
| **BD917** | *H.* sp. 01 | Sapal de Castro Marim / Rio Guadiana, Castro Marim | n.a. | 0 | 0 | 0 | 0 | 0 | 0 | 0 | 0 | Y |
| **BD918** | *H.* sp. 01 | Sapal de Castro Marim / Rio Guadiana, Castro Marim | n.a. | 0 | 0 | 0 | 0 | 0 | 0 | 0 | 0 | Y |
| **BD927** | *H. avicenniae* | Sapal de Castro Marim / Rio Guadiana, Castro Marim | n.a. | 0 | 0 | 0 | 0 | 0 | 0 | 0 | 0 | Y |
| **BD928** | *H. avicenniae* | Sapal de Castro Marim / Rio Guadiana, Castro Marim | n.a. | 0 | 0 | 0 | 0 | 0 | 0 | 0 | 0 | Y |
| **BD929** | *H. avicenniae* | Sapal de Castro Marim / Rio Guadiana, Castro Marim | n.a. | 0 | 0 | 0 | 0 | 0 | 0 | 0 | 0 | Y |
| **BD930** | *H. avicenniae* | Sapal de Castro Marim / Rio Guadiana, Castro Marim | n.a. | 0 | 0 | 0 | 0 | 0 | 0 | 0 | 0 | Y |

n.a. Not analysed, Y, amplified.
