# Supplementary material for: Marine Oomycetes of the Genus Halophytophthora Harbor Viruses Related to Bunyaviruses
Source: Front Microbiol. 2020 Jul 15;11:1467. doi: 10.3389/fmicb.2020.01467 (PMC7375090; doi:10.3389/fmicb.2020.01467)
Supplement: TABLE S2 — Mapping results for P. cinnamomi as reference sequence. [file Data_Sheet_1.docx]

Supplementary Material

**Table S2**. Mapping results for *P. cinnamomi* as reference sequence

| **#reads**  **(R1 +R2)** | **#mapped reads (R1+ R2)** | **% mapped reads** | **% multiple mapping** | **% mis** | **% proper** | **%single** | **%both** | **#HQlink** |
| --- | --- | --- | --- | --- | --- | --- | --- | --- |
| 358'922'078 | 114'709'252 | 31.96% | 0.13% | 3.11% | 30.54% | 1.32% | 30.64% | 13530 |

'#reads': number of reads in the library, '#mapped reads': number of primary alignments (for a read with multiple segments aligned, only the larger one is taken into account), '%mapped reads': '#mapped reads' / '#reads' * 100 Secondary: one of the many places a read can be aligned. Each aligned read has at least one primary and 0 or more such secondary alignments. '% multiple mappings': proportion of mapped segments matching several positions on the reference sequences (more exactly, this value is the number of mapped segments having a mapping quality < 4 divided by the number of mapped segments), '%mis': proportion of sequenced bases having a mismatch with the reference sequence

**Table S3.** Gene-Specific Primers (GSPs) used in the SMARTer RACE reactions.

| **Name** | **^P^Sequence 5'-3'** | **Contig end** | **N to the end of the contig*** | **Tm (°C)** |
| --- | --- | --- | --- | --- |
| Virus1_RACE1_FW | GGATCTAGAGTTCGCCGCTCGTG | 3´ | 121 | 65 |
| Virus1_RACE 1_RE | CCGGTCTCGTTCAACTCGGACAG | 5´ | 136 | 65.1 |
| Virus2_RACE1_FW | TTGCATAACAGCTGCCGCCCGGA | 3´ | 743 | 69.4 |
| Virus 2_RACE 1_RE | AGCTTTCGGGTCCCATGTTGGCA | 5´ | 405 | 67 |
| Virus3_RACE1_FW | CTGCTGCAAAGGGGAAAGGTGGCA | 3´ | 780 | 68 |
| Virus 3_RACE 1_RE | CGACCCACTTCCCGCTAGGTCCCT | 5´ | 332 | 69.4 |
| Virus  4_RACE1_FW | AGGTTCACATGGTTGGGCCCTAGC | 3´ | 899 | 66.8 |
| Virus 4_RACE 1_RE | AGGTCAGCAGGAATGTCCATGCCTT | 5´ | 540 | 66.3 |
| Virus5_RACE1_FW | TGGTCTACGTTCTCAAGCAATTCGGGCA | 3´ | 73 | 68.2 |
| Virus 5_RACE1_RE | CCCAGGGCAAGGAATCCGAACAGGGA | 5´ | 610 | 70 |
| Virus 6_RACE1_FW | ACAACGCAAGCTGCTCGGGCTGT | 3´ | 419 | 69.8 |
| Virus 6_RACE1_RE | ACAGAAAACGCAGGCCCCCTCGT | 5´ | 467 | 68.8 |
| Virus 7_RACE1_FW | ACCGTTGCAAGCCTTTGGTCAGCCA | 3´ | 252 | 69.6 |
| Virus 7_RACE1_RE | TGGTATTTGTTGCACCTGTTGATGT | 5´ | 704 | 61.7 |
| Virus 8_RACE1_FW | GTCTCCCGGACGGTTACAACTGCA | 3´ | 221 | 66.7 |
| Virus 8_RACE1_RE | CCCGCATGTCAACCTCACCATTTAGCA | 5´ | 1,027 | 67.4 |
| Virus8_RACE2_FW | TCGAGAGTGGCTGAAGCAGTTCGA | 3´ | 578 **^A^** | 65.9 |

**^P^** All the primers were ordered with 15 bp added to the 5´ end (GATTACGCCAAGCTT) as described by the manufacturer.

*Nucleotides from the site of the primer to the end in the original contig nucleotide sequence to the of the initial contig end (kb)

**^A^** Virus8_RACE2_FW was designed based on the virus sequence obtained after the first RACE. This is the distance to the end of the second contig obtained.

**Table S4.** Specific primers used for the partial amplification of the viral RdRp in order to screen the presence of each virus in all the *Halophytophthora* isolates of the collection.

| **Name** | **Sequence 5'-3'** | **PCR product size (bp)** | **^&^Annealing temperature (°C)** |
| --- | --- | --- | --- |
| Virus 1_FW | GCAAATCAGGGACTCAGGCT | 700 | 55 |
| Virus 1_RV | CATGCAGAAGATTTCGCGGG |  |  |
| Virus 2_FW | AGCTTCAGAGAATGTGGCCC | 500 | 55 |
| Virus 2_ RE | CCCAACGCCTACAATCCAGT |  |  |
| Virus 3_ FW | CGGCAAATTGCTCGATGAGG | 700 | 55 |
| Virus 3_ RE | ACCACTGTCATCGGAATGCG |  |  |
| Virus 4_ FW | CTCAGCTGCTCCGAGGTAAT | 700 | 55 |
| Virus 4_ RE | CCCAACAGGTGAAGGATGGT |  |  |
| Virus 5_ FW | TTTCCCTTTCACCAGTCGTCA | 700 | 55 |
| Virus 5_ RE | GCTATCATCGGAATGGGCCA |  |  |
| Virus 6_ FW | TGGGGTTCTGTGTTGCTGAA | 0.7 | 55 |
| Virus 6 _RE | CCGGGCTTCCTTTGACAGAT |  |  |
| Virus 7_FW | GTGTAACTGCTTCCTCAACTTGA | 0.7 | 54 |
| Virus 7 _RE | GAAACATCAACAGGTGCAACA |  |  |
| Virus 8.1 _FW | GTTTCGCTATGCCCATCACA | 0.7 | 53 |
| Virus 8.1_RE | CCATGTCTGCCCTGTTCCTT |  |  |
| MIDFWDACT | TGGCACCACACCTTCTACAA | 0.7 | 54 |
| MIDREVACT | GAACCACCGATCCAGACGGAGTA |  |  |

**^&^** Annealing temperature (°C) for each RT-PCR performed in each virus detection.

**Table S5.** Percentages of pairwise identities between the eight viral full-length nucleotide genome sequences.

|  | **HRV1** | **HRV7** | **HRV3** | **HRV2** | **HRV6** | **HRV5** | **HRV8** | **HRV4** |
| --- | --- | --- | --- | --- | --- | --- | --- | --- |
| **HRV1** |  |  |  |  |  |  |  |  |
| **HRV7** | 23.45 |  |  |  |  |  |  |  |
| **HRV3** | 32.26 | 27.52 |  |  |  |  |  |  |
| **HRV2** | 32.13 | 27.89 | 57.05 |  |  |  |  |  |
| **HRV6** | 27.42 | 27.92 | 34.34 | 35.76 |  |  |  |  |
| **HRV5** | 25.05 | 25.85 | 29.06 | 29.98 | 27.94 |  |  |  |
| **HRV8** | 24.69 | 25.26 | 33.31 | 33.34 | 30.06 | 26.41 |  |  |
| **HRV4** | 27.32 | 25.62 | 38.29 | 39.00 | 36.39 | 27.35 | 30.26 |  |

**Table S6.** Percentages of pairwise identities between the eight amino acid RdRp sequences.

|  | **HRV1** | **HRV7** | **HRV3** | **HRV2** | **HRV6** | **HRV5** | **HRV8** | **HRV4** |
| --- | --- | --- | --- | --- | --- | --- | --- | --- |
| **HRV1** |  |  |  |  |  |  |  |  |
| **HRV7** | 14.11 |  |  |  |  |  |  |  |
| **HRV3** | 14.60 | 15.26 |  |  |  |  |  |  |
| **HRV2** | 13.35 | 14.00 | 47.80 |  |  |  |  |  |
| **HRV6** | 11.77 | 15.88 | 17.13 | 18.25 |  |  |  |  |
| **HRV5** | 13.70 | 13.37 | 13.46 | 14.67 | 34.07 |  |  |  |
| **HRV8** | 9.76 | 9.00 | 10.45 | 9.75 | 11.77 | 10.28 |  |  |
| **HRV4** | 12.75 | 12.94 | 15.47 | 16.54 | 37.82 | 39.91 | 10.87 |  |
