# Supplementary material for: Marine Oomycetes of the Genus Halophytophthora Harbor Viruses Related to Bunyaviruses
Source: Front Microbiol. 2020 Jul 15;11:1467. doi: 10.3389/fmicb.2020.01467 (PMC7375090; doi:10.3389/fmicb.2020.01467)
Supplement: Supplementary file 3 [file Data_Sheet_2.docx]

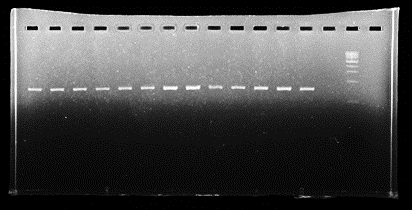


**- 0.5 kb**

**-1 kb**

**Figure S1.** Actin gene PCR products from left to right: amplified cDNA from 13 samples of *Halophytophthora* isolates, negative control and Quick-Load^®^ 1 kb Extend DNA Ladder (N3239S; NEB).
